# 3-[(3,4-Dichloro­phen­yl)amino­carbon­yl]propionic acid monohydrate

**DOI:** 10.1107/S1600536809024519

**Published:** 2009-07-01

**Authors:** B. Thimme Gowda, Sabine Foro, B. S. Saraswathi, Hartmut Fuess

**Affiliations:** a Department of Chemistry, Mangalore University, Mangalagangotri 574 199, Mangalore, India; b Institute of Materials Science, Darmstadt University of Technology, Petersenstrasse 23, D-64287 Darmstadt, Germany

## Abstract

In the crystal structure of the title compound, C_10_H_9_Cl_2_NO_3_·H_2_O, the conformations of the amide O atom and the carbonyl O atom of the acid segment are *anti* to the H atoms of adjacent –CH_2_ groups. In the crystal, the mol­ecules are linked into a three-dimensional network through N—H⋯O and O—H⋯O inter­molecular hydrogen bonds.

## Related literature

For related structures, see: Gowda *et al.* (2009**a* ▶,*b* ▶,c* ▶). For hydrogen bonds involving carboxylic acids, see: Jagannathan *et al.* (1994 ▶); Leiserowitz (1976 ▶). For the modeling of water H atoms, see: Nardelli (1999 ▶).
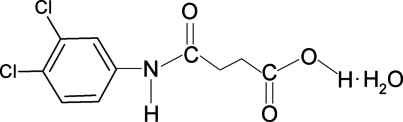


## Experimental

### 

#### Crystal data

C_10_H_9_Cl_2_NO_3_·H_2_O
*M* *_r_* = 280.10
Monoclinic, 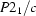

*a* = 9.5634 (9) Å
*b* = 7.4527 (7) Å
*c* = 17.292 (2) Å
β = 104.35 (2)°
*V* = 1194.0 (2) Å^3^
*Z* = 4
Cu *K*α radiation
μ = 4.95 mm^−1^
*T* = 299 K
0.55 × 0.50 × 0.40 mm

#### Data collection

Enraf–Nonius CAD-4 diffractometer
Absorption correction: ψ scan (North *et al.*, 1968 ▶) *T*_min_ = 0.098, *T*_max_ = 0.138
2508 measured reflections
2129 independent reflections
2052 reflections with *I* > 2σ(*I*)
*R*_int_ = 0.072
3 standard reflections frequency: 120 min intensity decay: 1.0%

#### Refinement

*R*[*F* ^2^ > 2σ(*F* ^2^)] = 0.083
*wR*(*F* ^2^) = 0.212
*S* = 1.08
2129 reflections
167 parameters
5 restraints
H atoms treated by a mixture of independent and constrained refinement
Δρ_max_ = 0.92 e Å^−3^
Δρ_min_ = −0.69 e Å^−3^

### 

Data collection: *CAD-4-PC* (Enraf–Nonius, 1996 ▶); cell refinement: *CAD-4-PC*; data reduction: *REDU4* (Stoe & Cie, 1987 ▶); program(s) used to solve structure: *SHELXS97* (Sheldrick, 2008 ▶); program(s) used to refine structure: *SHELXL97* (Sheldrick, 2008 ▶); molecular graphics: *PLATON* (Spek, 2009 ▶); software used to prepare material for publication: *SHELXL97*.

## Supplementary Material

Crystal structure: contains datablocks I, global. DOI: 10.1107/S1600536809024519/ci2838sup1.cif

Structure factors: contains datablocks I. DOI: 10.1107/S1600536809024519/ci2838Isup2.hkl

Additional supplementary materials:  crystallographic information; 3D view; checkCIF report

## Figures and Tables

**Table 1:** Hydrogen-bond geometry (Å, °)

*D*—H⋯*A*	*D*—H	H⋯*A*	*D*⋯*A*	*D*—H⋯*A*
O2—H2*O*⋯O4^i^	0.88 (3)	1.79 (3)	2.672 (4)	177 (5)
N1—H1*N*⋯O3^ii^	0.84 (3)	2.11 (3)	2.941 (4)	168 (4)
O4—H41⋯O1^iii^	0.82 (3)	2.11 (4)	2.894 (4)	162 (5)
O4—H42⋯O1^iv^	0.84 (3)	2.09 (3)	2.881 (4)	156 (5)

Symmetry codes: (i) 
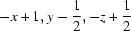
; (ii) 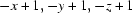; (iii) 
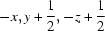
; (iv) 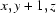.

**Fig. 1.**
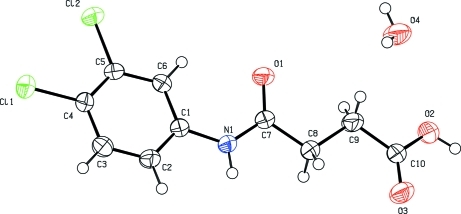
The molecular structure of the title compound, showing the atom labelling and the displacement ellipsoids are at the 50% probability level. H atoms are represented as small spheres of arbitrary radii.

**Fig. 2.**
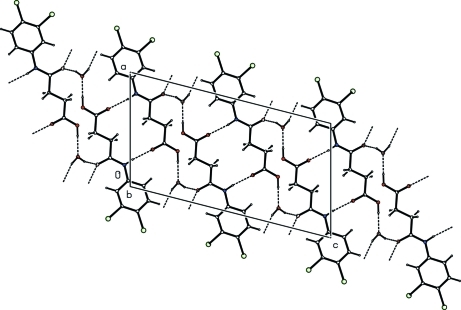
The crystal packing of the title compound, with hydrogen bonds shown as dashed lines.

## supplementary crystallographic information

### Comment

As a part of studying the effect of ring and side chain substitutions on the structures of aromatic amides (Gowda *et al.*, 2009*a,b,c*), the crystal structure of *N*-(3,4-dichlorophenyl)succinamic acid monohydrate (I), systematic name: 3-[(3,4-dichloro)-aminocarbonyl]propionic acid monohydrate has been determined. The conformation of the N—H bond is *anti* to both the 3-chloro substituent in the aromatic ring and the C═O bond in the amide segment of the structure. Further, the amide O atom and the carbonyl O atom of the acid segment are *anti* to each other and are also *anti* to H atoms attached to the adjacent C atoms (Fig.1). Further, C═O and O—H bonds of the acid group are *syn* to each other, contrary to the *anti* position observed in 3-[(3,5-dichloro)-aminocarbonyl]propionic acid (Gowda *et al.*, 2009*c*). The observed *anti* position with the latter may be due to the hydrogen bond donated to the amide carbonyl group by the acid segment, which is prevented in the present structure due to the H-bonding effect of hydration. The N—H···O and O—H···O intermolecular hydrogen bonds link the molecules into a three-dimensional network (Table 1 and Fig.2).

The modes of interlinking carboxylic acids by hydrogen bonds is described elsewhere (Leiserowitz, 1976). The packing of molecules involving dimeric hydrogen-bonded association of each carboxyl group with a centrosymmetrically related neighbor has also been observed (Jagannathan *et al.*, 1994).

### Experimental

The solution of succinic anhydride (0.02 mol) in toluene (25 ml) was treated dropwise with the solution of 3,4-dichloroaniline (0.02 mol) also in toluene (20 ml) with constant stirring. The resulting mixture was stirred for about 1 h and set aside for an additional hour at room temperature for the completion of reaction. The mixture was then treated with dilute hydrochloric acid to remove the unreacted 3,4-dichloroaniline. The resultant solid *N*-(3,4-dichlorophenyl)-succinamic acid was filtered under suction and washed thoroughly with water to remove the unreacted succinic anhydride and succinic acid. It was recrystallized to constant melting point from ethanol. The purity of the compound was checked by elemental analysis and characterized by its infrared spectra. Single crystals used in X-ray diffraction studies were grown in an ethanol solution by slow evaporation at room temperature.

### Refinement

The O-bound and N-bound H atoms were located in a difference map. The positional parameters of the N-bound H atom were refined with N-H = 0.86 (4) Å and those of the O-bound (hydroxyl) H atom were refined with O-H distance restrained to 0.82 (4) Å. The positions of water H atoms were refined with restrained geometry (Nardelli, 1999) *viz*. O-H = 0.85 (4) Å and H···H = 1.365 (4) Å. The other H atoms were positioned with idealized geometry using a riding model [C-H = 0.93–0.97 Å]. The isotropic displacement parameters of all H atoms were set to 1.2 times of the *U*_eq_ of the parent atom.

### Crystal data

**Table:**

C_10_H_9_Cl_2_NO_3_·H_2_O	*F*(000) = 576
*M**_r_* = 280.10	*D*_x_ = 1.558 Mg m^−^^3^
Monoclinic, *P*2_1_/*c*	Cu *K*α radiation, λ = 1.54180 Å
Hall symbol: -P 2ybc	Cell parameters from 25 reflections
*a* = 9.5634 (9) Å	θ = 4.8–20.7°
*b* = 7.4527 (7) Å	µ = 4.95 mm^−^^1^
*c* = 17.292 (2) Å	*T* = 299 K
β = 104.35 (2)°	Prism, colourless
*V* = 1194.0 (2) Å^3^	0.55 × 0.50 × 0.40 mm
*Z* = 4	

### Data collection

**Table:**

Enraf–Nonius CAD-4 diffractometer	2052 reflections with *I* > 2σ(*I*)
Radiation source: fine-focus sealed tube	*R*_int_ = 0.072
graphite	θ_max_ = 67.0°, θ_min_ = 4.8°
ω/2θ scans	*h* = −11→1
Absorption correction: ψ scan (North *et al.*, 1968)	*k* = −8→0
*T*_min_ = 0.098, *T*_max_ = 0.138	*l* = −19→20
2508 measured reflections	3 standard reflections every 120 min
2129 independent reflections	intensity decay: 1.0%

### Refinement

**Table:**

Refinement on *F*^2^	Secondary atom site location: difference Fourier map
Least-squares matrix: full	Hydrogen site location: inferred from neighbouring sites
*R*[*F*^2^ > 2σ(*F*^2^)] = 0.083	H atoms treated by a mixture of independent and constrained refinement
*wR*(*F*^2^) = 0.212	*w* = 1/[σ^2^(*F*_o_^2^) + (0.1684*P*)^2^ + 0.6399*P*] where *P* = (*F*_o_^2^ + 2*F*_c_^2^)/3
*S* = 1.08	(Δ/σ)_max_ = 0.003
2129 reflections	Δρ_max_ = 0.92 e Å^−^^3^
167 parameters	Δρ_min_ = −0.69 e Å^−^^3^
5 restraints	Extinction correction: *SHELXL97* (Sheldrick, 2008), Fc^*^=kFc[1+0.001xFc^2^λ^3^/sin(2θ)]^-1/4^
Primary atom site location: structure-invariant direct methods	Extinction coefficient: 0.014 (2)

### Special details

**Table:**

Geometry. All e.s.d.'s (except the e.s.d. in the dihedral angle between two l.s. planes) are estimated using the full covariance matrix. The cell e.s.d.'s are taken into account individually in the estimation of e.s.d.'s in distances, angles and torsion angles; correlations between e.s.d.'s in cell parameters are only used when they are defined by crystal symmetry. An approximate (isotropic) treatment of cell e.s.d.'s is used for estimating e.s.d.'s involving l.s. planes.
Refinement. Refinement of *F*^2^ against ALL reflections. The weighted *R*-factor *wR* and goodness of fit *S* are based on *F*^2^, conventional *R*-factors *R* are based on *F*, with *F* set to zero for negative *F*^2^. The threshold expression of *F*^2^ > σ(*F*^2^) is used only for calculating *R*-factors(gt) *etc*. and is not relevant to the choice of reflections for refinement. *R*-factors based on *F*^2^ are statistically about twice as large as those based on *F*, and *R*- factors based on ALL data will be even larger.

### Fractional atomic coordinates and isotropic or equivalent isotropic displacement parameters (Å^2^)

**Table:**

	*x*	*y*	*z*	*U*_iso_*/*U*_eq_	
Cl1	−0.35278 (8)	0.10598 (12)	0.56662 (5)	0.0441 (4)	
Cl2	−0.33021 (8)	0.03602 (12)	0.38996 (4)	0.0449 (4)	
O1	0.1190 (2)	0.2952 (3)	0.33626 (13)	0.0417 (6)	
O2	0.5845 (3)	0.4233 (4)	0.26373 (15)	0.0534 (7)	
H2O	0.674 (4)	0.448 (6)	0.262 (3)	0.064*	
O3	0.6264 (3)	0.5188 (4)	0.38871 (15)	0.0543 (7)	
N1	0.1755 (3)	0.3197 (3)	0.47092 (15)	0.0331 (6)	
H1N	0.240 (4)	0.353 (5)	0.511 (2)	0.040*	
C1	0.0452 (3)	0.2667 (4)	0.48923 (17)	0.0301 (7)	
C2	0.0368 (3)	0.2923 (4)	0.56697 (18)	0.0345 (7)	
H2	0.1138	0.3446	0.6036	0.041*	
C3	−0.0840 (3)	0.2416 (4)	0.59077 (18)	0.0367 (7)	
H3	−0.0879	0.2582	0.6435	0.044*	
C4	−0.2005 (3)	0.1652 (4)	0.53626 (17)	0.0319 (7)	
C5	−0.1905 (3)	0.1364 (4)	0.45895 (17)	0.0315 (7)	
C6	−0.0693 (3)	0.1877 (4)	0.43433 (17)	0.0325 (7)	
H6	−0.0646	0.1696	0.3818	0.039*	
C7	0.2060 (3)	0.3302 (4)	0.39971 (17)	0.0311 (7)	
C8	0.3601 (3)	0.3889 (4)	0.40446 (18)	0.0360 (7)	
H8A	0.3773	0.5034	0.4319	0.043*	
H8B	0.4260	0.3022	0.4359	0.043*	
C9	0.3923 (4)	0.4068 (7)	0.3247 (2)	0.0566 (11)	
H9A	0.3294	0.4973	0.2939	0.068*	
H9B	0.3717	0.2938	0.2963	0.068*	
C10	0.5465 (3)	0.4577 (5)	0.33053 (19)	0.0400 (8)	
O4	0.1421 (3)	0.9875 (5)	0.23932 (16)	0.0570 (8)	
H41	0.070 (4)	0.945 (6)	0.209 (3)	0.068*	
H42	0.116 (5)	1.059 (6)	0.271 (3)	0.068*	

### Atomic displacement parameters (Å^2^)

**Table:**

	*U*^11^	*U*^22^	*U*^33^	*U*^12^	*U*^13^	*U*^23^
Cl1	0.0340 (5)	0.0587 (6)	0.0425 (6)	−0.0077 (3)	0.0149 (4)	0.0020 (3)
Cl2	0.0329 (5)	0.0645 (7)	0.0332 (6)	−0.0134 (3)	0.0003 (4)	−0.0034 (3)
O1	0.0306 (11)	0.0668 (15)	0.0259 (11)	−0.0049 (10)	0.0035 (9)	−0.0020 (9)
O2	0.0391 (13)	0.0936 (19)	0.0297 (13)	−0.0109 (13)	0.0123 (10)	0.0014 (12)
O3	0.0375 (13)	0.0839 (19)	0.0395 (14)	−0.0159 (13)	0.0058 (11)	−0.0119 (13)
N1	0.0270 (12)	0.0455 (14)	0.0256 (13)	−0.0077 (10)	0.0045 (10)	−0.0018 (10)
C1	0.0263 (13)	0.0343 (14)	0.0291 (15)	0.0006 (10)	0.0057 (11)	0.0034 (11)
C2	0.0326 (15)	0.0411 (15)	0.0279 (15)	−0.0056 (12)	0.0039 (12)	−0.0048 (11)
C3	0.0392 (16)	0.0438 (16)	0.0281 (15)	−0.0024 (12)	0.0103 (12)	−0.0045 (12)
C4	0.0273 (14)	0.0380 (14)	0.0310 (14)	−0.0010 (11)	0.0085 (11)	0.0037 (11)
C5	0.0265 (14)	0.0372 (14)	0.0272 (14)	−0.0027 (11)	−0.0001 (11)	0.0022 (11)
C6	0.0313 (14)	0.0417 (16)	0.0237 (14)	−0.0022 (11)	0.0055 (11)	0.0017 (11)
C7	0.0255 (14)	0.0359 (14)	0.0301 (14)	0.0002 (11)	0.0039 (11)	0.0017 (11)
C8	0.0299 (15)	0.0489 (17)	0.0288 (16)	−0.0071 (12)	0.0063 (12)	0.0003 (12)
C9	0.0322 (18)	0.110 (3)	0.0274 (17)	−0.0167 (18)	0.0062 (13)	−0.0016 (17)
C10	0.0315 (16)	0.060 (2)	0.0281 (15)	−0.0061 (13)	0.0071 (12)	0.0040 (13)
O4	0.0364 (13)	0.094 (2)	0.0387 (14)	−0.0004 (13)	0.0064 (11)	−0.0163 (14)

### Geometric parameters (Å, °)

**Table:**

Cl1—C4	1.723 (3)	C3—H3	0.93
Cl2—C5	1.726 (3)	C4—C5	1.381 (4)
O1—C7	1.229 (4)	C5—C6	1.384 (4)
O2—C10	1.319 (4)	C6—H6	0.93
O2—H2O	0.88 (3)	C7—C8	1.520 (4)
O3—C10	1.193 (4)	C8—C9	1.492 (5)
N1—C7	1.337 (4)	C8—H8A	0.97
N1—C1	1.415 (4)	C8—H8B	0.97
N1—H1N	0.84 (3)	C9—C10	1.501 (4)
C1—C2	1.380 (4)	C9—H9A	0.97
C1—C6	1.390 (4)	C9—H9B	0.97
C2—C3	1.372 (4)	O4—H41	0.82 (3)
C2—H2	0.93	O4—H42	0.84 (3)
C3—C4	1.390 (4)		
			
C10—O2—H2O	118 (3)	C1—C6—H6	120.5
C7—N1—C1	128.9 (2)	O1—C7—N1	123.8 (3)
C7—N1—H1N	117 (3)	O1—C7—C8	122.8 (3)
C1—N1—H1N	114 (3)	N1—C7—C8	113.4 (3)
C2—C1—C6	119.8 (3)	C9—C8—C7	113.3 (3)
C2—C1—N1	116.5 (3)	C9—C8—H8A	108.9
C6—C1—N1	123.6 (3)	C7—C8—H8A	108.9
C3—C2—C1	120.7 (3)	C9—C8—H8B	108.9
C3—C2—H2	119.6	C7—C8—H8B	108.9
C1—C2—H2	119.6	H8A—C8—H8B	107.7
C2—C3—C4	120.2 (3)	C8—C9—C10	112.6 (3)
C2—C3—H3	119.9	C8—C9—H9A	109.1
C4—C3—H3	119.9	C10—C9—H9A	109.1
C5—C4—C3	118.9 (3)	C8—C9—H9B	109.1
C5—C4—Cl1	121.4 (2)	C10—C9—H9B	109.1
C3—C4—Cl1	119.6 (2)	H9A—C9—H9B	107.8
C4—C5—C6	121.3 (3)	O3—C10—O2	123.7 (3)
C4—C5—Cl2	120.6 (2)	O3—C10—C9	124.5 (3)
C6—C5—Cl2	118.1 (2)	O2—C10—C9	111.8 (3)
C5—C6—C1	119.0 (3)	H41—O4—H42	109 (4)
C5—C6—H6	120.5		
			
C7—N1—C1—C2	−171.2 (3)	C4—C5—C6—C1	1.2 (4)
C7—N1—C1—C6	10.6 (5)	Cl2—C5—C6—C1	−179.2 (2)
C6—C1—C2—C3	−0.3 (5)	C2—C1—C6—C5	0.1 (4)
N1—C1—C2—C3	−178.6 (3)	N1—C1—C6—C5	178.3 (3)
C1—C2—C3—C4	−0.8 (5)	C1—N1—C7—O1	1.1 (5)
C2—C3—C4—C5	2.1 (5)	C1—N1—C7—C8	−178.6 (3)
C2—C3—C4—Cl1	−178.8 (3)	O1—C7—C8—C9	2.4 (5)
C3—C4—C5—C6	−2.3 (4)	N1—C7—C8—C9	−177.9 (3)
Cl1—C4—C5—C6	178.6 (2)	C7—C8—C9—C10	−177.7 (3)
C3—C4—C5—Cl2	178.1 (2)	C8—C9—C10—O3	−16.9 (6)
Cl1—C4—C5—Cl2	−1.0 (4)	C8—C9—C10—O2	161.8 (3)

### Hydrogen-bond geometry (Å, °)

**Table:**

*D*—H···*A*	*D*—H	H···*A*	*D*···*A*	*D*—H···*A*
O2—H2O···O4^i^	0.88 (3)	1.79 (3)	2.672 (4)	177 (5)
N1—H1N···O3^ii^	0.84 (3)	2.11 (3)	2.941 (4)	168 (4)
O4—H41···O1^iii^	0.82 (3)	2.11 (4)	2.894 (4)	162 (5)
O4—H42···O1^iv^	0.84 (3)	2.09 (3)	2.881 (4)	156 (5)

Symmetry codes: (i) −*x*+1, *y*−1/2, −*z*+1/2; (ii) −*x*+1, −*y*+1, −*z*+1; (iii) −*x*, *y*+1/2, −*z*+1/2; (iv) *x*, *y*+1, *z*.
